# What we need as we get older: needs assessment for the development of a community geriatrics service in an Australian context

**DOI:** 10.1186/s12877-021-02553-8

**Published:** 2021-10-25

**Authors:** Mark I. Hohenberg, Najwa-Joelle Metri, Rubab Firdaus, David Simmons, Genevieve Z. Steiner

**Affiliations:** 1 School of Medicine, Western Sydney University, Penrith, NSW 2751 Australia; 2 NICM Health Research Institute, Western Sydney University, Locked Bag 1797, Penrith, NSW 2751 Australia; 3 School of Health Science, Western Sydney University, Penrith, NSW 2751 Australia; 4 Campbelltown Hospital, South Western Sydney Local Health District, Campbelltown, NSW 2560 Australia; 5 Translational Health Research Institute (THRI), Western Sydney University, Locked Bag 1797, Penrith, NSW 2751 Australia

**Keywords:** Community geriatrics service (CGS), Needs assessment, Aged care, Older people, Qualitative

## Abstract

**Background:**

The aim of this study was to inform the development of a Community Geriatrics Service (CGS) that addressed the healthcare and social needs of community dwelling older people in an Australian context.

**Methods:**

Stakeholders (*N* = 108) took part in a ‘needs assessment’ involving 30-min semi-structured interviews with general practitioners (GPs; *N* = 49), and three 2-h focus groups (community engagement meetings; *N* = 59) with older people, informal caregivers, allied healthcare workers, and nursing home directors. Data were transcribed and thematically coded, mapped to source and weighted to the frequency that the theme was raised across sources.

**Results:**

Five themes informing CGS development and delivery emerged: active health conditions (management of behavioural and psychological symptoms of dementia, falls, multimorbidity, and other relevant conditions), active social challenges (patient non-compliance, need for aged care social workers, caregiver stress, elder abuse, social isolation, and stigma), referrals (availability of specialists, communication, specialist input, and advance care directives), access (lack of transport options, and inaccessibility of local geriatrics clinics and specialists), and awareness (lack of awareness, knowledge, and resources).

**Conclusions:**

The CGS will need to address access, referral processes and health system navigation, which were perceived by stakeholders as significant challenges. These findings warrant the development of a CGS with an integrated approach to aged care, pertinent for the health and social needs of the elderly.

## Background

Worldwide, the number of people aged 60 years and older has doubled over the past 30 years [1]. Similar to other industrialised countries, Australia is experiencing significant population ageing, with more than one in every seven people aged 65 years and over in 2017 [1]. Population ageing is driving a rise in Australia’s old-age dependency ratio [1, 2], and this is largely attributable to a 3.3% increase in the population aged 65 years and over (1996–2016) [3]. It is expected that there will be over 6.4 million older Australians by 2051; nearly triple the ~ 2.3 million in 1999 [4].

The rate of population ageing presents many challenges for federal, state, and local planners [5]. In Australia, population ageing is expected to result in a loss of 0.4% in revenue and add 0.3% to spending by 2028–2029, equating to a $36 billion annual cost, exceeding the anticipated total cost of Medicare in the same timeframe [1]. These increased costs are largely associated with the greater level of assistance required for day-to-day activities in order to maintain functional independence required by approximately 10% of people aged 65 to 74 years, and over 50% of people aged over 85 years [1]. These proportions compare with only 3% of individuals aged less than 65 years [1].

A Community Geriatrics Service (CGS) aims to improve access to specialist geriatric healthcare for older people requiring management for complex conditions, through outreach into general practice with case review or joint consultation [6]. CGSs provide overall support through geriatrician outreach, consultation, the promotion of multidisciplinary team care, and management plans that focus on delaying or reducing the likelihood of hospital or residential care admissions [6, 7]. A focus on community and informal care effectively maintains the functional independence of individuals [8]. For these reasons, CGSs have been developed globally to address both specific and generalised challenges in elderly populations [9]. In Australia, current CGS models of care support patients at home, in residential aged care, or in an outpatient setting and may be complete services aimed at preventing acute hospital presentation [10], or specific services such as wound or skin care [11].

Identifying and addressing gaps in service provision can inform the strategy for local service providers, improve the healthcare of the local population, reduce the burden on local hospitals, and can lead to new and improved models of care that can be translated nationally and internationally. Providing community-based care for older individuals will improve the diagnosis, treatment, and management of chronic conditions in an appropriate manner that is tailored to the social and cultural needs of the community [8], whilst decreasing healthcare spend [12]. The present study aimed to gain a holistic understanding of the social and healthcare requirements of community dwelling older people in South Western Sydney (SWS), where individuals aged 65 years and over account for 11.9% of the population [13]. A needs assessment was conducted to identify and understand gaps in current service provision, with the purpose of developing an integrated approach to aged care via a CGS.

## Methods

### Study design and setting

The needs assessment employed a qualitative study design to inform the development of a new CGS within SWS Local Health District (SWSLHD). This approach was taken for three reasons: 1) previous local studies (unpublished data) employing quantitative questionnaire methods had low response rates and did not adequately capture data of sufficient validity to inform operational outcomes; 2) the qualitative design provided an opportunity for rich (and potentially unexpected) data to inform the evolving CGS; and 3) it facilitated a co-creation approach that engaged prospective CGS end-users to ensure the model of care was relevant, whilst enhancing the potential for its utilisation once established.

Data sources included transcripts from semi-structured interviews with general practitioners (GPs) and digital audio recordings of focus groups with community healthcare and allied health professionals, older people and their caregivers and representatives (referred to as community engagement meetings). Across March–June 2016, data were collected at 3 participating sites in the local government areas (LGAs) comprising the Macarthur region of SWS, NSW, Australia: Camden, Campbelltown, and Wollondilly, which are peri-urban, urban, and rural, respectively.

### Recruitment and participants

Invitations were sent from March–June 2016 to primary care practices and allied health workers, nursing home directors, and patient and carer representative groups. The recruitment process was coordinated by the (male) community geriatrician (MIH) via SWS Primary Health Network (SWSPHN), Camden Council, Campbelltown City Council, Wollondilly Shire Council, and contacts through Campbelltown and Camden Hospitals within SWSLHD. The recruitment strategy for primary care practices included cold calling a non-exhaustive list of GP practices provided in-kind by CJU Medical Marketing. Using a purposive sampling strategy, invitations were open to a broad range of individuals. For this reason, controlling for factors such as cultural background, gender, age, and years of experience was not possible and not within the scope of this quality assurance (QA) project. Informed consent was verbally implied from all participants prior to commencing interviews and community engagement meetings. This project was a QA activity that did not require Human Research Ethics Committee approval and was conducted with appropriate oversight as detailed in the NHMRC’s Ethical Considerations in Quality Assurance and Evaluation Activities document.

### Data collection and analyses

Semi-structured interviews up to 30 min in length were conducted for each GP, and three 2-h community engagement meetings were facilitated by the community geriatrician (who is experienced in qualitative research interviewing). A standard set of questions was used across all interviews and focus groups (see Table 1). Participants were engaged to ascertain their understanding of local healthcare service provision, and to identify the needs of older people and caregivers in the region. From this, priority areas for future healthcare provision planning were identified.

**Table 1:** Semi-structured interview (GPs) and focus group (community engagement meeting) schedule

Section	Topics Discussed	Interview Duration	Focus Group Duration
Introductions	Meet and greet	1 min	5 min
Explanation of background to development	Explain the nature of the study, the role of geriatricians, and the basic principles of the objectives of the planned service	2 min	5 min
Consent	Check for any questions and obtain verbal consent to participate	1 min	5 min
Access demand for CGS	1. How many geriatric patients do you see daily approximately? 2. How many geriatric patients have 2 or more major active health problems? 3. Do these active health problems include diabetes? 4. How many geriatric patients have active social issues? 5. Do your patients experience any difficulties with driving?	5 min	25 min
Requirements	1. What are the hardest medical issues your geriatric patients have? 2. What are the hardest social issues your geriatric patients have? 3. Who are your hardest geriatric patients? 4. Is there anything that your geriatric patients need that they are not getting? 5. Do you feel there is a need for more advanced care directives? 6. How many patients would you refer to secondary care? 7. Are there some patients you would like to refer but are unable so, and if so, why? 8. What would you envisage geriatricians helping you and your patients with?	5 min	25 min
Geriatricians	1. What would your expectations of a CGS be? 2. What are your issues with the current geriatric service? 3. What would be your priority requirements from a CGS? 4. Would you prefer to have an on-call geriatrician, one geriatrician or a team of geriatricians allocated to your practice?	5 min	25 min
Conclusions	1. Is there anything else you feel is important to highlight at this stage?	2 min	15 min

All interview responses were directly transcribed by the community geriatrician during the interview, while focus groups were digitally recorded and summarised by two independent non-contributing attendees, and later transcribed. The transcripts were analysed via the open axial coding method [14] and thematically coded by a single independent research analyst (RF) using Quirkos v.1.3 computerised qualitative analysis software [15]. Data were then mapped to source and weighted to the frequency that the theme was raised across sources. The iterative analysis process consisted of identifying and coding the data to ascertain topical responses and emergent substantive categories, coding for word repetition, direct and demanding statements, and discourse markers including intensifiers, connectives, and evaluative clauses. Emerging categories among the codes allowed for the development of several themes and sub-themes. All transcripts were initially coded by one researcher (RF), reviewed by a second researcher (MIH), and then thematically organised by the wider research team (MIH, NJM, GZS).

## Results

### Sample characteristics and data sources

Interviews were conducted with 49 GPs. The community engagement meeting held in Camden included 28 representatives, Wollondilly, 15, and Campbelltown, 16. Collectively, these representatives were from 4 residential aged care facilities (RACFs) and various health service providers and support organisations including Liverpool Hospital Aged Care Services, Ambulance Service, Campbelltown City Council, Wollondilly Shire Council, National Seniors Macarthur, Longevity Senior Services, Dementia Advisory Service, and Dementia Australia (see Table 2). Approximately 4337 older people are cared for by these service providers each day: RACFs care for 1476 residents; the GPs see 695 older people, and the health service providers see 2166 individuals.

**Table 2:** Participant demographics for each of the three community engagement meetings

Focus Group	No. of Participants
***Camden***	
RACF Resident	16
Organisation/Service Representative	6
RACF Staff	4
Government Representative	1
University Staff	1
***Campbelltown***	
Organisation/Service Representative	10
Community-Dwelling Older Adult	3
Government Representative	2
University Staff	1
***Wollondilly***	
Organisation/Service Representative	9
Community Representative	2
Community-Dwelling Older Adult	2
University Staff	1
RACF Staff	1

### Qualitative data

Five overarching themes emerged. Theme 1, *Active Health Conditions*, included four sub-themes: management of challenging behaviours in dementia, management of falls, management of multimorbidity, and other relevant conditions. Theme 2, *Active Social Challenges*, included six sub-themes: patient non-compliance, need for aged care social workers, caregiver stress, elder abuse, social isolation, and stigma. Theme 3, *Referrals*, included two sub-themes: availability of specialists and communication, and specialist input and advance care directives. Theme 4, *Access*, included two sub-themes: lack of transport options, and inaccessibility of local geriatrics clinics and specialists. Theme 5, *Awareness*, included three sub-themes: lack of awareness, lack of knowledge, and lack of resources. All statistics presented within these themes were described by study participants relating to their specific organisation, and may not be generalisable to other service providers across the sector.

#### Theme 1: active health conditions

When asked about active health conditions, representatives of three RACFs indicated that the most common and difficult to manage medical condition amongst older people is dementia. Specifically, the behavioural and psychological symptoms of dementia (BPSD) were discussed as the most challenging due to their complex and demanding nature, for example,*“behavioural and psychological symptoms of dementia are some of the hardest medical issues geriatric patients have, which require more staff to look after.”* ~ RACF 1.

Providers indicated that falls occur frequently. RACF 2 reported 22 falls in a month, of which 5 residents were hospitalised. RACF 3 indicated that 1–6 residents are referred to secondary care due to falls, bleeding, and/or loss of consciousness, expressing, *“some hard-medical issues include falls and malnutrition. Doctors [are] not available for 24 hours. For falls, review by doctors would help.”* Findings indicate that onsite falls assessment is an important service gap relevant to the CGS that could lead to reduced hospitalisations.

Consumers and community representatives described many relevant health conditions that could be addressed by the CGS or other local health policies and services. Further, GPs indicated that many older patients experience multiple active chronic medical conditions, with 82% of geriatric patients managed by GPs experiencing two or more active health conditions. The lack of available podiatrists was highlighted as a major issue, with GP referrals not acted upon in a timely manner, or at all. The demand for podiatric services corresponded with the high rates of diabetes amongst older people residing in RACFs in all three LGAs; 35% of residents and 41% of GPs’ geriatric patients were estimated to have a diagnosis.

Dental care was also of particular concern. When residents or geriatric patients presented with dental conditions, they were often directed to hospitals, rather than dentists. Within the Campbelltown LGA, people with dementia were reported to experience dental conditions disproportionately. Community representatives have indicated the need for more convenient and subsidised access to dentistry. Mental health conditions were reported to be highly prevalent and problematic amongst the older population. Participants from Camden expressed dissatisfaction with long waiting lists, and a lack of public mental health services within the region. Malnutrition within rural and peri-urban communities was also described, which may be relevant to the high rates of falls in those communities.

#### Theme 2: active social challenges

GPs reported active social challenges amongst 57% of geriatric patients, and RACFs described these amongst less than 10% of residents. Non-compliance was the most common challenge and barrier to care, where patients refuse placements and referrals, particularly people with dementia experiencing challenging behaviours. One GP stated, for example, “*deconditioned, non-compliant patients and frequent attendees … refusing placement, patients refuse to go to care.”* ~ GP 1. Most participants indicated that patient non-compliance with treatment plans leads to disadvantaged health outcomes, demonstrating a need for specialised communication. GPs and RACF providers expressed that the development of an aged care social work service would improve communication and prevent the referral of individuals to hospitals to access these services, for instance, *“need [for] geriatric social work leads to sending patients to hospitals to access social work.”* ~ GP 2.

Consumers and community representatives described high levels of stress amongst caregivers of older people. High levels of caregiver stress were attributed to social pressures and difficulty dividing their time between work and care. Many caregivers considered their caring role as a responsibility to family members; this was particularly true within culturally and linguistically diverse (CALD) communities with strong family ties.

Elder abuse and social isolation were identified as significant social challenges requiring urgent attention. Participants thought that elder abuse was becoming more prevalent within the Macarthur region, and that the community would benefit from the distribution of resources to raise awareness of elder abuse. Social isolation in rural and peri-urban areas of Camden and Wollondilly was thought to be a consequence of the geographic location and a lack of accessible transport.

Social stigma was considered a barrier to effective service utilisation. Consumers and community representatives felt that stigma contributed to the under-diagnosis of illnesses, ailments, and chronic conditions, such as dementia, particularly so for older men who have reported not seeing a physician in years. Patient and caregiver groups discussed the need for education within the community to eradicate the social stigma surrounding illnesses and remove these personal barriers to accessing care, “*education is required for the community, including the elderly and GPs, regarding this, to break the stigma.” ~* Community Representative 1. Across groups, most participants shared the view that education is necessary to create a culture of normalcy regarding healthcare and ageing; this is an important challenge that the CGS could address.

#### Theme 3: referrals

Participants reported that geriatric patients and residents experiencing acute illness, including falls, are often referred to secondary care. However, RACF representatives expressed the view that GPs share different perspectives on when to refer a patient to a specialist: *“some GPs have a lower understanding of when to refer to the specialist and … there is a resistance to refer. This will significantly impact on the quality of care provided.”* ~ RACF 1. These data suggest that communication between primary and secondary care providers lacked transparency and could hinder care quality, reinforcing the need for streamlined, integrated, multidisciplinary care. Despite the view that GPs lacked the understanding of when to refer patients, most of the GPs interviewed discussed their expectations of geriatricians regarding the management of long-term cases, with GP 4 stating, *“geriatricians need to help troubleshoot new or chronic problems to help aged conditions.”* GPs articulated their expectation that geriatricians should help with mobility, motor and/or functional conditions, implying a willingness to collaborate.

There was a demand for comprehensive geriatric assessment of complex cases. The GPs interviewed indicated that they were not aware what services were offered by specialists, and that there were barriers to effective communication between GPs and specialists. Community Representative 2 explained, *“integration is a huge part of this, between primary [care] to hospitals and back, a lot of work needs to be done … they’re not talking to each other so the result for the patient is that the information is not flowing as freely.”* A common view shared by most community representatives was that older people experience disadvantaged health outcomes and high levels of hospital referrals due to fragmented care. When asked about the need for specialist input, many participants indicated the need for advance care directives in various environments, particularly areas that have low socio-economic status, amongst CALD populations, and in RACFs.

#### Theme 4: access

When asked about challenges regarding access to facilities and aged care services, many participants indicated that a lack of transport options was a significant issue. Community Representative 3 stated, *“the issue with access is logistic[s]… with these clinics, people need a way to get to them…”* A key contributory factor was ability to drive, with 23% of GPs (caring for 695 geriatric patients daily) expressing concerns about older people driving. GPs reported that driving was challenging for their patients, and this would be an increasing issue in the future. The concern surrounding transport extended across all three LGAs, but was a greater issue for participants from rural and semi-rural areas. Affordability was also identified as a barrier for accessing specialist services, as well as the documentation involved in the process. The cost of specialist healthcare consultation was a greater issue for rural and semi-rural participants. Together, findings suggest the need for reduced-cost remote care options to combat accessibility issues.

#### Theme 5: awareness

When asked about guardianship, consumers and community representatives from Camden and Wollondilly LGAs indicated that the larger population was unaware of the way in which enduring guardianship of older people should be established. There were similar concerns regarding power of attorney, where GP 7 expressed *“more advance [care] directives are required in low socioeconomic areas.”* Indeed, GPs were not aware of the difference between an advance care directive, which primarily focuses on future healthcare needs, and power of attorney, which relates to financial decision-making in the event of lost capacity.

Consumers and representatives suggested that there was a lack of resources available regarding active health and social challenges faced by older people within the community. Participants stated that education would be the most effective method to address these issues. Suggestions included a community outreach program as an influential action plan for the dissemination of information. Participants felt that these resources should be aimed toward the children, spouses, and other informal caregivers of older people, and should include a specific focus on advance care planning.

## Discussion

This project captured stakeholder insights on the barriers to geriatric service use experienced by the Macarthur community. Findings highlight the perceived importance of a novel CGS that addresses the health and social challenges of older people, facilitates access to services and resources, and addresses referral processes and health system navigation. These barriers and social challenges are well-documented in multiple settings in the literature, for instance, in diabetes care [16].

Managing BPSD, falls, and multimorbidity is challenging in the community care setting. The development of an in-home geriatric programme in the Netherlands found that such programmes can improve the detection of symptoms and management of BPSD [17]. The risk of falls among older adults can be mitigated through balance training programs [18], virtual reality training [19], and dynamic posture training [20], which are all potential avenues for CGS service augmentation. Additionally, a world-leading BPSD service established in Australia has helped improve the management of BPSD in RACFs through multimodal psychosocial interventions, which may be adopted by CGS programmes [21]. Multimorbidity was also reported as a concern, which is somewhat unsurprising, given that SWS is thought to be affected by significantly disproportionate health outcomes compared to greater Sydney, including higher prevalence of diabetes, cardiovascular disease (CVD), and lung, gastro-intestinal, liver, kidney, and thyroid cancers [22–25]. Costs in managing multimorbidity in the community setting can be reduced through an effective CGS. For example, in the USA, the per patient cost of at-home case-managed individuals is valued at approximately 75% of the cost of institutional care [12].

Active social challenges included non-compliance with treatment plans, which may be exacerbated by the cognitive deficits associated with dementia. Additional social challenges are also associated with BPSD [26]. CGS development must consider early diagnosis of cognitive impairment and the interactions between cognitive deficits and treatment management [26], as well as the effective and timely dissemination of this information to stakeholders [6]. Gerontological social workers may also relieve communication difficulties, given that they are trained to conduct holistic geriatric assessments [27], create conducive housing environments, and encouraging treatment-adherence [28]. However, there are currently no gerontological educational programs available across all undergraduate fields in Australia and New Zealand, unlike the USA which offers gerontology-specific degrees at all educational levels [29, 30]. Further, it is thought that social work curricula are missing a strong gerontology focus, despite the critical role that social workers could play in the management of older peoples’ health in the community [31, 32].

Caregiver stress was highlighted as a major challenge within SWS, where primary caregivers often experience poor health and negative health behaviours [33]. Caregiver stress is more prominent in CALD populations, where there are varying degrees of functional dependency such as language barriers that may attribute to high levels of caregiver stress and elder abuse [34, 35]. This is also observed in Aboriginal and Torres Strait Islander communities, who experience higher rates of depression, coupled with economic and social disadvantage, when compared to the general population [36]. A CGS may relieve these stressors by referring caregivers to support and respite services, whilst providing tailored advice for kinship caregiving. Moreover, elder abuse is prevalent in all cultural groups within society; one in four older adults are estimated to be vulnerable to elder abuse [37]. Community services, including the CGS, may hence form an essential component of the strategy for preventing and stopping elder abuse, through the identification and referral of at-risk individuals to the relevant support systems.

Social isolation within SWS was of concern, and has previously been identified as an independent risk factor for low levels of self-reported health and wellbeing [38]. Self-perceived loneliness has been linked with increased risk of chronic conditions, including dementia [39], and all-cause mortality [40]. Social isolation has been exacerbated by social distancing interventions during the COVID-19 pandemic [40]. Purposeful social engagement may be an effective public health intervention for socially excluded members of the community [38]; an area where the CGS could play a role. Age-related social stigma was described as a challenge as it can lead to marginalisation and the under-diagnosis of illnesses affecting older people, including dementia [41]. Age-related stigma is associated with a ‘hypercognitive culture,’ which results in conditions including dementia being viewed as discreditable, ultimately isolating and marginalising older people [41]. Social stigma is also exacerbated in CALD communities, where the challenging behaviours in dementia are thought to cause feelings of fear within the community [42]. The lack of public awareness associated with social stigma extends to non-age related diseases, including diabetes [16]. By providing informal and community care, CGS programmes may play an essential role in destigmatising chronic conditions prevalent amongst older adults.

Poor integration between primary and secondary care creates several issues. In some areas, 64–68% of primary and secondary care doctors believe that patient care is not well-coordinated between their two levels [43]. In the context of diabetes management in SWSLHD, stakeholders state that traditional modes of collaboration between primary and secondary care professionals lack transparency, as communication lines are typically closed and the processes lack visibility [44]. Australian primary care services are also thought to have poor linkages with other relevant services, and more training is required for primary healthcare providers to understand the multidisciplinary needs of specific age groups [45]. Improving communication, information sharing, and providing a model of effective digital health using multidisciplinary healthcare teams within a CGS is considered to be one of the most effective approaches for community patients to access improved consistency of care, also known as integrated care [43].

Advance care directives were deemed as very important due to their ability to improve individuals’ and caregivers’ quality of life, quality of dying, and drive the establishment of advance care plans [46]. Providing targeted education and training to healthcare practitioners and RACF staff on advance care planning allows an opportunity to ameliorate the disproportionate health outcomes and high levels of caregiver stress experienced in SWS [22]. Low levels of understanding of enduring guardianship and power of attorney have been linked with a lack of knowledge regarding medico-legal services available to older adults and their caregivers [16, 47]. This knowledge gap can be overcome through community-directed action plans to raise awareness of the health challenges faced by older people. A CGS is well-placed to deliver such an action plan.

Limited community transport has been described elsewhere, for example, in New Zealand, where loss of existing transport options may threaten the functional independence and quality of life of older adults [48]. In Australia, mobility and health challenges create substantial barriers to public transport usage, and driving cessation as a consequence of these challenges is often linked to a perceived reduction in independence [49]. Through CGS development, multidisciplinary healthcare teams can travel to the patient, diminishing accessibility issues. A well-resourced CGS allowing multi-disciplinary review in the community will help to address accessibility issues.

Given the COVID-19 pandemic, virtual health or telehealth has also become increasingly utilised to deliver quality healthcare [50]. By mainstreaming this method of care and providing the healthcare workforce with the appropriate accreditation and training, telehealth options may relieve many of the logistical and equity issues surrounding healthcare access [50]. The inclusion of virtual health in CGS development may contribute to improved health outcomes for the local community through increasing the accessibility and reach of the programme. Virtual health, and the reimbursements provided by the Medicare Benefits Schedule (MBS), a selection of healthcare services subsidised by the Australian government, may also mitigate the financial burden of specialist geriatric services. Specifically, three MBS items allow for the comprehensive health assessment of older people to identify any health risks, as well as a broad range of factors which influence social, psychological, and physical wellbeing [51]. Under the MBS, a rebate is payable once annually for each eligible patient [51]. The affordability of the CGS is paramount, as participants in similar studies have expressed that specialist geriatric services should be free of cost or have minimal out-of-pocket expenses to ensure service utilisation [52].

This project had several strengths, including data triangulation and a relatively large sample size for a qualitative study. However, the investigation was designed to inform service development; thus, data saturation was not considered crucial to this outcome, and subsequently, was not achieved, which may compromise the validity of results. Resources permitting, future work could explore a broader range of issues across a larger geographical area with a greater sample size, and indeed, such a study is underway. Potential introductions of bias, which could have affected the reliability of the data, include the use of a single researcher to conduct the interviews, varying transcription methods, and the use of a finite list of primary care providers provided by the medical marketing company. The primary outcome of this needs assessment was to inform the development of a local service; this was achieved. The results were highly informative and guided the successful development of a CGS in SWS. This paper, the methods used, and the findings may be valuable for other groups when assessing community needs, providing a scalable method to inform the development of CGS programmes relevant to local community needs. An evaluation of the CGS will be conducted in due course.

## Conclusion

This QA project highlighted many gaps in gerontological service provision in SWS, including health and social challenges, as well as issues pertaining to referrals, access to healthcare, and awareness of the services provided locally. These findings confirmed the necessity for the development of community services tailored to the health and social issues of older people, and informed the successful development of a CGS in SWS. Services that are increasing clinical effectiveness whilst also reducing costs are the cornerstone of contemporary clinical and healthcare economic practice; a CGS is a good example of what can be achieved in this regard. Furthermore, if appropriate local research on community needs is conducted, a new local CGS is likely to be more efficacious and utilised by relevant stakeholders for the benefit of the local population, clinically, socially, and economically.

## Data Availability

The datasets generated and analysed during the current study are not publicly available because data are qualitative in nature (recorded interviews and focus groups that were transcribed verbatim) and could potentially result in the reidentification of participants. Deidentified transcripts are available from the corresponding author on reasonable request.

## Abbreviations

CGS: Community Geriatrics Service
SWS: South Western Sydney
SWSLHD: South Western Sydney Local Health District
SWSPHN: South Western Sydney Primary Health Network
LGA: Local Government Area
GP: General Practitioner
QA: Quality Assurance
RACF: Residential Aged Care Facility
CALD: Culturally and Linguistically Diverse
CVD: Cardiovascular Disease
COVID-19: Coronavirus Disease
BPSD: Behavioural and Psychological Symptoms of Dementia
